# The Effects of Total Knee Arthroplasty on Sleep Quality

**DOI:** 10.5704/MOJ.1907.002

**Published:** 2019-07

**Authors:** M Koken, B Guclu

**Affiliations:** Department of Orthopaedics and Traumatology, Ufuk University, Ankara, Turkey

**Keywords:** total knee arthroplasty, sleep quality, Pittsburgh scale

## Abstract

**Introduction:** Symptomatic osteoarthritis is one of the most common indications for total knee arthroplasty (TKA) operations. Pain in every stage of the disease causes sleep disturbances in patients. The primary objective of this study was to evaluate the effect of TKA on the quality of sleep in patients with symptomatic osteoarthritis.

**Materials and Methods:** This retrospective, descriptive study was performed on 80 patients diagnosed with symptomatic osteoarthritis who underwent TKA. The patients responded to the Pittsburgh Sleep Quality Index (PSQI), which is widely used to evaluate sleep quality. Additionally, the effects of demographic and clinical variables such as age, gender, body mass index, smoking and alcohol consumption were also evaluated before and after surgery.

**Results:** There was no correlation between demographic variables and PSQI scores pre- and postoperatively. There was a decrease in sleep quality on the sixth postoperative week compared to the preoperative period however this difference was not statistically significant. On the other hand, there was a statistically significant difference between preoperative and postoperative sixth month PSQI scores. Mean values of overall sleep quality and daily function were significantly higher in the postoperative sixth compared to the preoperative period (p<0.001)

**Conclusion:** Treatment of symptomatic OA with TKA will improve sleep quality in the long term.

## Introduction

Total knee arthroplasty (TKA) is a widely utilised surgical treatment of symptomatic osteoarthritis of the knee (OA). Symptomatic OA is associated with a low quality of life (QOL) due to chronic pain and decreased ability to perform daily activities^1,2,3^. On the other hand, QOL is directly related to sleep quality. There are conflicting reports on the effects of TKA on sleep quality in the literature^4,5^.

Although there are different methods for the evaluation of sleep quality in adults, the Pittsburgh Sleep Quality Index (PSQI) is globally used. The PSQI which is a self-reporting and easily understood questionnaire, was devised by Buysee *et al* based on the evaluation of sleep quality over a one month time interval^6^. Although this questionnaire is widely used in studies, there is a paucity of studies conducted on patients treated with TKA for symptomatic OA.

The primary objective of this study was to assess the effect of TKA on sleep quality in treatment of osteoarthritis. The patients were evaluated at three time intervals: preoperative, postoperative 6th week and 6th month. We also investigated the effects of related variables such as age, body mass index, sex, smoking, alcohol consumption and duration of symptoms.

## Materials and Methods

This descriptive observational study was conducted between January 2015 and March 2017 on 80 patents who had undergone TKA (71.25% female) between 57-75 years of age and were followed up for at least six months after the surgery. The study was approved by the Institutional Ethics Committee and conducted in accordance with the Helsinki Declaration. Written informed consent was obtained from all patients. Patients who had undergone bilateral TKA, revision knee arthroplasty or unicondylar knee arthroplasty were excluded from the study in order to prevent a non-homogenous study population. Height, weight and waist circumference (WC) were measured and body mass index (BMI) was calculated. The frequency of alcohol consumption was recorded as daily, more than two days a week, every two weeks, once a month, rarely or none. None of the patients had a history of sleep apnea, sleep disorders or treatment for sleep disturbances.

Sleep quality was evaluated using the PSQI which was developed by Buysee *et al* in 1989. This questionnaire is a self-reporting tool which includes 19 items which generate seven components such as subjective sleep quality, sleep latency, sleep duration, habitual sleep efficiency, daytime dysfunction, sleep disturbances and medicines used for sleep. The maximum score for these subdimensions is 21. A higher score indicates a worse sleep quality^7^. The scores were calculated by free online software (https://psqicalculator.bracketglobal.com/). The PSQI was applied one week before the operation, six weeks and six months after the operation.

Visual Analogue Score (VAS) was measured simultaneously with the PSQI for all patients. The VAS score is determined by measuring the distance (mm) on a 10 cm line representing a spectrum from “no pain” (VAS=0) and “the worst pain” (VAS=10). Based on the distribution of VAS scores in postsurgical patients (knee replacement, hysterectomy or laparoscopic myomectomy) who described their pain intensity as none, mild, moderate or severe, the following cut-off points on the pain VAS were recommended: no pain (0-4 mm), mild pain (5-44 mm), moderate pain (45-74 mm) and severe pain (75-100 mm)^8^. Pain intensity was recorded preoperatively and on the postoperative sixth week and sixth month as reported by the patients based on perceived pain intensity and effect on daily activities.

Data analyses were performed using SPSS for Windows, version 22.0 (SPSS Inc., Chicago, IL, United States). Whether the distribution of continuous variables was normal or not was determined by the Kolmogorov Smirnov test. Levene test was used for the evaluation of homogeneity of variances. Unless specified otherwise, continuous data were described as mean ± SD for normal distributions, and median (range) for skewed distributions. Categorical data were described as number of cases (%).

Statistical analysis differences in normally distributed variables between two independent groups were compared by Student’s t test, and Mann Whitney U test was applied for comparisons of the abnormally distributed data. While the differences in normally distributed variables among more than two independent groups were analysed by One-Way ANOVA, otherwise, Kruskal Wallis test was applied for comparisons of the abnormal data.

## Results

The study population consisted of TKA patients whose characteristics were as follows: average age was 63.8 ± 6.5 years, duration of symptoms, including pain, was 4.6 ± 3.3 years, 71.25% were female and all were postmenopausal. History of alcohol consumption was a follows: none 92.5%, rarely 2.5%, once in a month 1.25%, every two weeks 2.5% and daily 1.25%. Only 13 (16.25%) patients were smokers. Eight (10%) patients were obese/overweight (BMI > 90. percentile). Forty-five patients were operated on the left knee while 35 patients were operated on the right.

Spinal or epidural anesthesia was administered in all operations. A tourniquet was placed in flexion which was deflated after bone cement hardening. An anterior midline incision with the knee in flexion was used for skin incision. Medial parapatellar approach was used. Osteophytes were removed by performing osteotomies on the femur and tibia. Zimmer-Biomet cemented Vanguard^®^ posterior stabilised total knee prosthesis was implanted for all patients. The patella was shaped using a rongeur instrument without patellar resurfacing. The articular capsule, soft tissue and skin was closed with the knee in 90^o^ flexion. The negative pressure drainage was removed after 24 hours and the patients could perform full weight-bearing walk.

All patients walked with a walker and full weight-bearing, used a continuous passive motion device (CPM) two times a day with the range of motion 0^o^ extension and 90^o^ flexion. After three weeks, the operated knee was flexed at 110-120^o^ and the patients was able to walk independently.

All patients were discharged to home with no change in daily activities after a four-week recovery period. Clinical characteristics of the participants are presented in Table I.

**Table I:** Demographic and clinical data of participants

			Patients (n=80)	
Age (y)^a^	63.8±6.5			
Body mass index (kg/m^2^)	<25	(n=56)		
	>25	(n=8)		
Sex	F	(n=57)		
	M	(n=23)		
Smoker	10.4%	(n=13)		
Alcohol consumption	None		: 92.5%	(n=74)
	Daily		: 0.8%	(n=1)
	Every 2 week		: 1.6%	(n=2)
	Monthly		: 0.8%	(n=1)
	Rarely		: 0.8%	(n=2)
Left / Right	59 / 21			

^a^ Values are reported in mean ± SD

Reported VAS scores were as follows: 41.7 ± 7.8 (mild-moderate pain) in the preoperative period, 35.6 ± 11.2 (mild-moderate pain) on the sixth postoperative week and 24.8 ± 9.2 (mil-moderate pain) on the sixth postoperative month. Both pain intensity and the PSQI scores revealed a statistically significant change on the postoperative sixth month compared to the preoperative period (p < 0.05). VAS scores showed a concordant decrease on the sixth postoperative month compared to the preoperative period and sixth postoperative week (p < 0.05). The PSQI scores on the sixth postoperative week were higher than the preoperative scores, although the difference was not statistically significant. There was a worsening in sleep disturbance and daytime dysfunction subdimensions, which could be directly related to the recovery period. There was a statistically significant difference between preoperative period and the sixth postoperative month concerning the PSQI scores. Overall sleep quality and daytime dysfunction scores were significantly higher on the postoperative sixth month compared to the preoperative period (p < 0.001). Table II summarises the PSQI results.

**Table II:** Total and subdimensional PSQI scores of participants

	Preop	Postop 6th week	Postop 6th month
Duration Of Sleep	0.4 ± 0.6	0.3 ± 0.4	0.7 ± 0.5
Sleep Disturbance	1.3 ± 0.7	1.6 ± 1.0	1.4 ± 0.9
Latency	0.8 ± 0.5	0.8 ± 0.8	0.7 ± 0.9
Day Dysfunction	1.2 ± 0.6	1.4 ± 0.8	0.6 ± 0.7
Efficiency	0.5 ± 0.4	0.8 ± 0.4	0.4 ± 0.6
Overall Sleep Quality	1.2 ± 0.8	1.1 ± 0.7	0.9 ± 0.8
Medications	0.8 ± 0.5	1.0 ± 1.3	0.6 ± 0.6
**Total PSQI**	**7.1 ± 3.5**	**7.4 ± 6.1**	**5.2 ± 2.1**

There was no statistically significant relationship between the baseline and postoperative PSQI scores and age, gender, BMI, alcohol consumption and smoking.

Data showed that TKA has positive effects on sleep quality based on PSQI changes. TKA has its main benefits on day dysfunction and sleep quality subdimensions.

## Discussion

The rise in the number of TKA procedures has caused a parallel rise in the number of studies on the effects of surgery on the QOL. While sleep disturbances are one of the main factors contributing to a low QOL, it is worse with osteoarthritis. Studies have revealed that sleep disturbances are more common than expected in the healthy population and investigations of the preoperative and postoperative period of TKA have shown that sleep quality varies after the surgery^7,9^. Studies have also revealed sleep disturbances in the early weeks and months following TKA^10^. However, these studies are lacking in terms of comparison of sleep quality between the pre- and postoperative periods.

Our data shows that sleep quality is negatively affected in the preoperative period. The preoperative PSQI score was 7.1 ± 3.5. This means that the sleep quality of patients with OA is impaired. However, the worst quality of sleep was observed on the postoperative sixth week, which can be explained as being during the recovery period. This can also be explained by pain due to the operation because most of the patients were able to perform daily activities at the fourth or sixth weeks following surgery. The fact that VAS scores show a positive correlation with the PSQI scores seems to an indisputable result of the effect of pain on sleep.

When the subgroups are compared, there is a statistically significant increase in sleep quality at the sixth postoperative month (p < 0.05). Total PSQI scores at this time were lower than the baseline, indicating a better sleep quality. Manning *et al*^7^ reported a transient sleep disturbance in the early postoperative period with an improvement above the preoperative baseline at ten months after primary TKA. These results show that late postoperative evaluation for sleep disturbances using the PSQI is more meaningful.

The difference was statistically significant concerning day dysfunction and overall sleep quality between baseline and six months (p < 0.05). Surprisingly, we detected no changes in sleep disturbances, sleep latency and efficacy. Demographic and clinical data of the participants were also compared for PSQI subscores and no statistically significant differences were found.

Identifying sleep disturbances and quality in adults is not easy. This study was based on the hypothesis that sleep quality would be lower in patients with osteoarthritis compared to the healthy population, due to pain, decreased activity and old age. There are other additional factors that could adversely affect sleep quality such as gastroesophageal reflux, migraine, arrhythmias and chronic obstructive lung disease. Further studies should be designed to compare for concomitant diseases, which was a limitation of our study. Another limitation of this study was the fact that results were not compared with a healthy population of the same age. Finally, we did not evaluate additional functional and pain scales of the patients.

This study illustrates that further studies evaluating the effect of aggressive pain treatment in symptomatic OA and the early postoperative period are necessary. One of the major limitations of this study was not evaluating other factors which directly affect sleep quality. Future studies should focus on the comparison of surgical treatments with medical treatments or different types of surgical treatments on sleep quality.

## Conclusion

Total knee arthroplasty is a proven method for the treatment of pain and loss of function in symptomatic osteoarthritis. Treatment of symptomatic OA with TKA will improve sleep quality in the long term. One of the most important criteria for optimal treatment is the effect on quality of life.
